# Temperature Field Boundary Conditions and Lateral Temperature Gradient Effect on a PC Box-Girder Bridge Based on Real-Time Solar Radiation and Spatial Temperature Monitoring

**DOI:** 10.3390/s20185261

**Published:** 2020-09-15

**Authors:** Xiao Lei, Xutao Fan, Hanwan Jiang, Kunning Zhu, Hanyu Zhan

**Affiliations:** 1 College of Civil and Transportation Engineering, Hohai University, 1 Xikang Road, Nanjing 210098, China; leix2010@126.com (X.L.); fanxutao11@163.com (X.F.); 2 Civil & Environmental Engineering Department, University of Wisconsin Platteville, Platteville, WI 53818, USA; 3 China Design Group Co., Ltd., 9 Ziyun St., Nanjing 210014, China; redkun@163.com; 4 Klipsch School of Electrical and Computer Engineering, New Mexico State University, Las Cruces, NM 88003, USA; hzhan@nmsu.edu

**Keywords:** lateral temperature gradient, temperature field, boundary conditions, solar radiation intensity, temperature monitoring

## Abstract

Climate change could impose great influence on infrastructures. Previous studies have shown that solar radiation is one of the most important factors causing the change in temperature distribution in bridges. The current temperature distribution models developed in the past are mainly based on the meteorological data from the nearest weather station, empirical formulas, or the testing data from model tests. In this study, a five-span continuous Prestressed-concrete box-girder bridge was instrumented with pyranometers, anemometers, strain gauges, displacement gauges, and temperature sensors on the top and bottom slabs and webs to measure the solar radiation, wind speeds, strain, displacement, and surface temperatures, respectively. The continuously monitoring data between May 2019 and May 2020 was used to study the temperature distributions caused by solar radiation. A maximum positive lateral temperature gradient prediction model has been developed based on the solar radiation data analysis. Then, the solar radiation boundary condition obtained from the monitoring data and the lateral temperature gradient prediction model were utilized to compute the tensile stresses in the longitudinal and transverse directions. It was demonstrated in this study that the tensile stress caused by the lateral temperature gradient was so significant that it cannot be ignored in structural design.

## 1. Introduction

Most highway bridges are located in open fields and are exposed to the solar radiation, wind, rain, and other environmental changes. Those climate changes can cause substantial temperature variations in bridges, trigger cracking and bearing displacing, and therefore, endanger the serviceability, durability, and safety of the bridges. In extreme cases, the major structural elements could lose their functionality due to the severe damage causing the global structural failure [1,2]. Solar radiation plays an important role in affecting the temperature distribution in the bridge, so that relationship between the solar radiation and the temperature distribution should be properly established. Lee [3] was monitoring the temperature distribution in a five-foot-long prestressed I-shape girder with thermocouples embedded in the flanges and the web for a year. With the continuously measured temperature variations, both vertical and lateral temperature distributions were established. A two-dimensional heat-transfer model was developed to investigate the effects of seasonal variations and girder orientations on temperature distributions. Wang et al. created a finite element model for a concrete box-girder arch bridge in thermal field caused by solar radiation based on meteorological data [4]. Abid et al. monitored air temperature, solar radiation, and wind speeds with sensors and thermocouples for more than a year. They proposed empirical equations to predict the maximum vertical and lateral temperature gradients based on the data collected [5]. The same approach was applied to concrete-encased steel girders [6]. Taysi and Abid investigated the effects of the thermal properties in a full-scale concrete box-girder segment. The individual effect of each of the thermal properties of the concrete box girder were studied with the aid of three-dimensional finite element analysis. Temperature distributions under the extreme thermal loads were compared with AASHTO’s (The American Association of State Highway and Transportation Officials) and Bridge Manual’s temperature gradient models [7]. Tian et al. conducted a numerical study on temperature effects in the train–bridge interaction system [8]. Lawson et al. used recent meteorological data from two weather stations in Nevada establishing temperature profiles for bridge superstructures and compared the results with AASHTO model [9]. Hagedorn et al. built an I-beam segment and determined vertical and transverse temperature gradients with temperature-monitoring data [10]. Rodriguez et al. implemented a dense array of thermocouples in a box-girder bridge in California and established a Finite Element model to predict bridge internal stresses under the measured temperature variations [11].

In the above-mentioned literature, either meteorological data from the nearest weather station or empirical equations were utilized to develop the temperature distribution resulting from solar radiation. However, oftentimes, the nearest weather station is still far from the bridge, so the meteorological data obtained could still deviate from the local climate conditions. Empirical equations were developed based on the given temperature data, hence they may not be applicable for a different on-site situation. Some studies used pyranometers to measure the intensity of solar radiation and thermocouples to measure the temperature distributions [5,6,7]. However, only the radiation intensity on the top flanges was investigated, while the effect of the flange shadow casting on the web was not discussed. In reality most box-girder bridges have tilted webs instead of vertical ones. Hence, to study the temperature distributions in box-girder bridges, it is essential to account for the tilt angle of the web and the overhang shadow casting on the web when estimating the solar radiation intensity.

In this study, we instrumented a prestressed concrete box-girder bridge with pyranometers, anemometers, strain gauges, displacement gauges, and temperature sensors on the top and bottom slabs and webs to measure the solar radiation, wind speeds, strain, displacement, and surface temperatures, respectively. The aforementioned data have been collected continuously for over a year and exploited to establish the solar radiation and temperature gradient relationship. In addition, a three-dimensional finite element model was developed to simulate the temperature field in the worst scenario and analyze the corresponding stress distribution based on the real time radiation monitoring data. The novelty of the work lies in the fact that all the boundary conditions have been obtained from real-time monitoring of solar radiation, temperature, and wind speed on an in-service bridge. From the data analysis, the lateral temperature gradient effect has been studied, which has long been ignored in the bridge design practice worldwide. Finally, the finite element analysis has been performed in conjunct with the maximum lateral temperature gradient from the monitoring data. The result indicated that the tensile stresses generated by the lateral temperature gradient alone are so significant that it cannot be ignored in design.

## 2. Experimental Instrumentation and Data Acquisition

**Figure 1:**
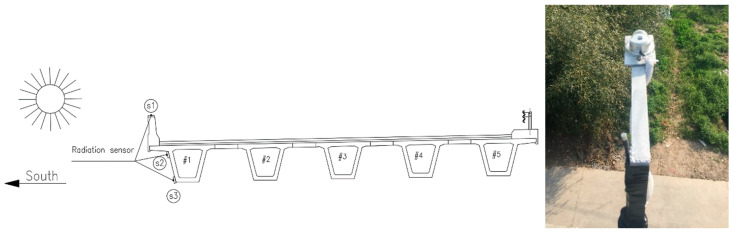
Radiation sensor (optical pyranometer) positions at the midspan.

**Figure 2:**
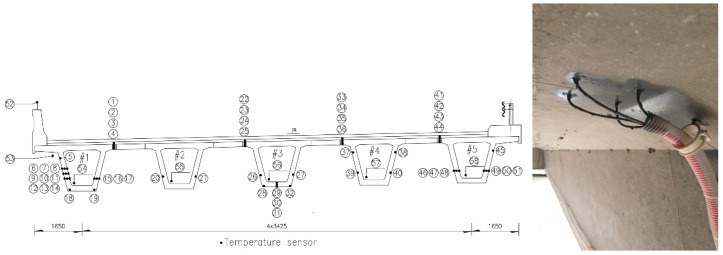
Temperature sensor layout at the midspan.

**Figure 3:**
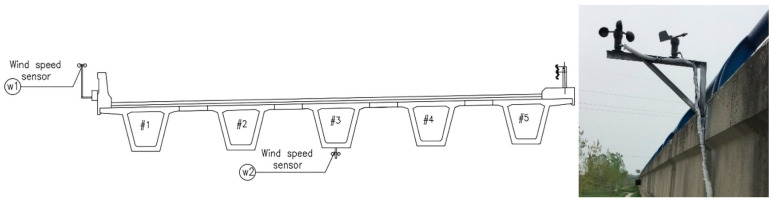
Anemometers for wind speed monitoring positions.

The bridge was built in 2002. It has five continuous spans and each span is 25 m in length. All the sensors were mounted on the fourth span from the south on upstream side. Three optical pyranometers (JMFS-1001) were installed on the top slab, upper corner on the web, and lower position on the web, respectively, for each bridge (Figure 1). The optical pyranometers have spectral range of 0.3~3 μm and can detect the direct radiation from the sun and the reflected radiation from the other objectives as well as the radiation from the incident sunlight with an angle. 58 temperature sensors were embedded inside or on the surface of the box girder in the midspan cross section (Figure 2). Two anemometers for wind speed monitoring were installed on the outer side and at the bottom of the bridge, as shown in Figure 3.

A comprehensive monitoring system was integrated with an all sealed chassis (JMBV-1164), an integrated data acquisition module (JMZX-32A), a temperature acquisition module (JMWT-64RT), and a DTU mobile internet module (JMTX-2017). The system enables 24/7 automatic continuous data acquisition through wire or wireless transmission. The data used in this study were collected from May 2019 to May 2020 at a sampling rate of 1 time per hour.

## 3. Solar Radiation and Heat Transfer Theory

The Fourier heat transfer differential equation was employed to describe the heat conduction in the concrete bridge [12,13,14,15]:

(1) $$\rho C_{p}\frac{\partial T}{\partial t}=\frac{\partial }{\partial x}(k\frac{\partial T}{\partial x})+\frac{\partial }{\partial y}(k\frac{\partial T}{\partial y})+\frac{\partial }{\partial z}(k\frac{\partial T}{\partial z})$$

where *ρ*, *C_p_*, and *k* are density in kg/m^3^, specific heat in J/kg °C, and thermal conductivity of concrete W/m °C, respectively. *T* refers to the temperature at any point in the girder at any time *t*.

The boundary condition on the surfaces of the box girder can be written as [12,13,14,15]:

(2) $$k\frac{\partial T}{\partial x}n_{x}+k\frac{\partial T}{\partial y}n_{y}+k\frac{\partial T}{\partial z}n_{z}+q_{c}+q_{s}+q_{re}=0$$

where *n_x_*, *n_y_*, and *n_z_* are the direction cosines of the normal vectors; *q_c_*, *q_s_*, and *q_re_* are the convection, the total solar radiation and the long-wave radiation in W/m^2^. The solar radiation *q_s_* consists of three components including direct solar radiation *q_dr_*, solar scattered radiation *q_sr_*, and ground reflected radiation *q_gr_*, as shown in Equation (3).

(3) $$q_{s}=q_{dr}+q_{sr}+q_{gr}$$

### 3.1. Direct Solar Radiation

Direct solar radiation comes straight from the Sun. The direct solar radiation energy *I_m_* can be expressed in the form of Equation (4) [13]:

(4) $$I_{m}=I_{0}\frac{\sin h}{\sin h+\frac{1-p}{p}}$$

where *h* is solar altitude angle; *p* is atmospheric transparency coefficient, and *I_0_* is solar constant.

Solar altitude angle *h* is generally written as Equation (5) [16].

(5) sin(h)=cosφcosδcosτ+sinφsinδ

where *ϕ* is the latitude of the location; *δ* is solar declination, and δ = 23.45 sin [360 (284 + N)/365] in which N is the day of the year; *τ* is the solar hour angle, which is zero at the noon, negative before noon, and positive afternoon converting each hour to 15°.

The direct solar radiation projected on the box-girder web *q_dr_* in Equation (3) is calculated as:

(6) $$q_{dr}=I_{m}\cos\theta$$

where *θ* is the angle between the Sun rays and the normal line to the web surface and cos*θ =* cos*β* sin*h +* sin*β* cos*h* cos(*γ_z_ − γ*) in which *β* is the angle between web and the horizontal direction, *γ* is the surface azimuth angle, and *γ_z_* is the azimuth angle of the sun.

In a box-girder bridge, the overhang casts shadow on the web and the solar radiation in the shadow should be deducted from the total. The equation of shadow length is defined by Equation (7) [16]:

(7) $$l_{s}=l_{c}\frac{\tan h}{\sin\left(90+\gamma -\gamma_{z}\right)\sin\beta -\cos\beta \tan h}$$

where *l_c_* is the length of the overhang.

### 3.2. Scattered Solar Radiation

Solar heat scatters on the structures in all directions. Jain assumed that the scattering intensity of the horizontal plane received is linearly related with the transmission coefficient and the averaged radiation. Scattering radiation *I_d_* in the atmosphere can be obtained with Equation (8) [17]:

*I_d_ = (*1* − *1.13* k_T_) I_m_* (8)

where *k_T_* is the transmission coefficient accounting for the attenuation of solar radiation in the atmosphere, which takes a value in between 0.3~0.8 for latitudes of 30~40°.

The scattered solar radiation *q_sr_* received by an arbitrary wall is:

*q_sr_ = I_d_ (*1* + cos β)/*2** (9)

### 3.3. Ground Reflected Radiation

The bottom slab of the box girder receives the short-length wave radiation reflected from the ground. The reflected solar radiation can be calculated by Equation (10) [18]:

(10) $$q_{gr}=\rho^{*}\left(I_{m}+I_{d}\right)\left(1-cos\beta \right)/2$$

where ρ* is the ground reflection coefficient and ρ* = 0.1 for ground reflection and ρ* = 0.2 for water reflection.

## 4. Theoretical and Measured Solar Radiation Intensity

**Figure 4:**
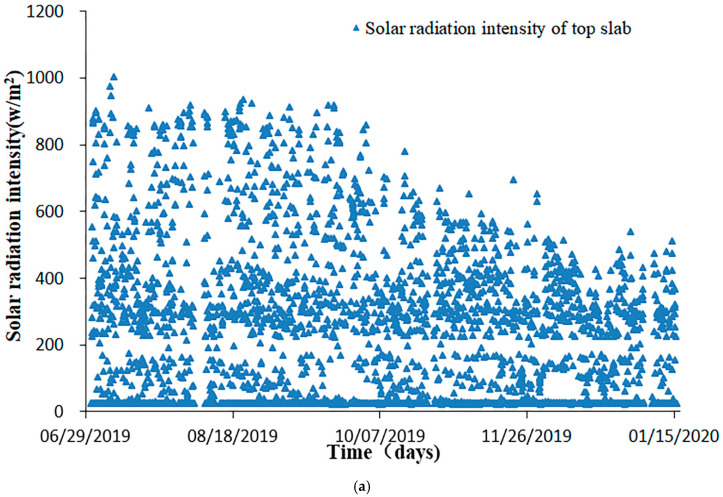

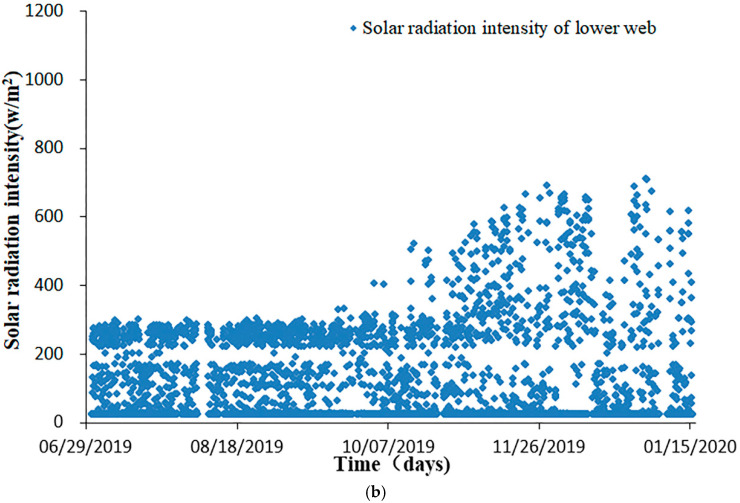
Solar radiation intensity on different parts of the bridge (sampling frequency: 1 time/h). (**a**) Solar radiation variations on the top slab from 29 June 2019 to 15 January 2020; (**b**) solar radiation variations on the lower web from 29 June 2019 to 15 January 2020.

It is evident from the monitoring data shown in Figure 4 that the solar radiation intensity on the top slab reached maximum in summer due to the direct solar exposure, while the intensity on the lower web reached maximum in winter. Solar radiation intensity on the lower web had maximum value in winter is because the top slab casts shadow on the web and the sun altitude angle gets smaller leading to higher solar radiation in winter.

**Figure 5:**
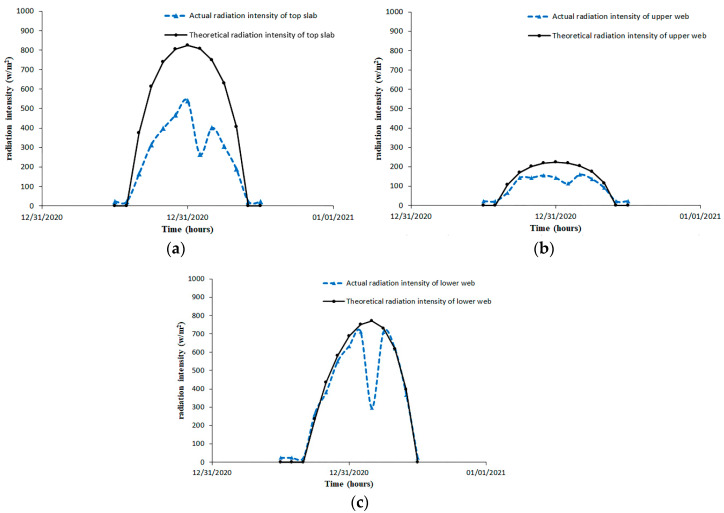
Comparison between measured and theoretical solar radiation intensities. (**a**) Solar radiation on the top slab; (**b**) solar radiation on the upper web; (**c**) solar radiation on the lower web.

From the aforementioned solar radiation theory, the theoretical solar radiation intensity was calculated and compared with the measured intensity, as shown in Figure 5. During the day of 31 December 2019, the measured solar radiation intensities on the top slab and upper portion of the web were smaller than the theoretical ones, while the measured solar radiation intensities on the lower portion of the web were close to the theoretical values. In addition, note that in Figure 5a,c there was a sudden drawdown around 2 pm, which may be attributed to the temporary cloudiness. The other reason for the underestimation of the theoretical equations may ascribe to the ignorance of the atmosphere counter radiation and earth surface radiation at night.

## 5. Solar Radiation and Temperature Gradient Relationship

The box-girder surface temperature increases rapidly under the solar radiation forming a large temperature gradient. The maximum daily temperature gradients were obtained from maximum temperature during the day at #12, #13, and #14 sensor subtracted by the lowest temperature at #15, #16, #17, #20, #21, #26, #27, #39, #40, #46, #47, #48, #49, #50, and #51. It was found that the daily maximum lateral temperature gradient was correlated with daily maximum solar radiation. The Pearson correlation coefficient for each location on the box girder was calculated, as shown in Table 1.

**Figure 6:**
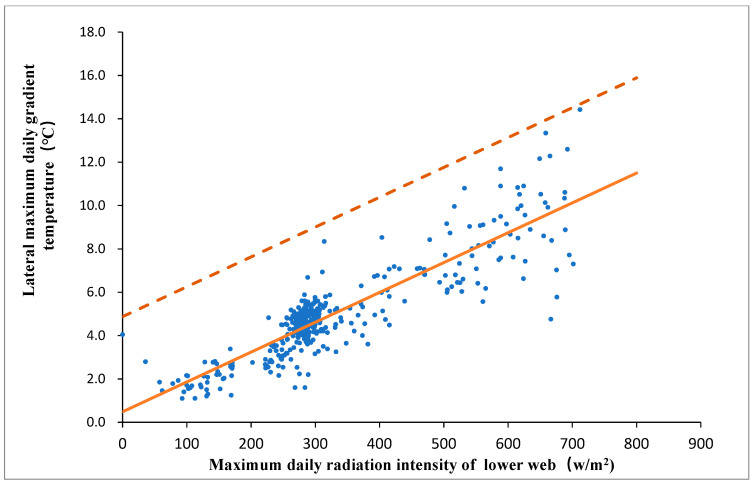
Relation between maximum daily positive lateral temperature gradient and maximum daily solar radiation intensity on the lower web.

It is indicated in Table 1 that the correlation between maximum daily positive lateral temperature gradient and maximum daily solar radiation on the lower web is the highest. In Figure 6, a linear regression was utilized to best fit the data described in Equation (11).

(11) $$T_{\;\max\text{-}\mathrm{la}}^{+}=0.01377I_{\max\text{-}\mathrm{lw}}+0.4838$$

where $T_{\;\max\text{-}\mathrm{la}}^{+}$ is the maximum daily positive lateral temperature gradient in °C and $I_{\max\text{-}\mathrm{lw}}$ is the maximum daily solar radiation intensity on the lower web. The standard deviation of $T_{\;\max\text{-}\mathrm{la}}^{+}$ d is equal to 2.196 °C. The dashed line in Figure 6 represents the $T_{\;\max\text{-}\mathrm{la}}^{+}+2d$, and it is just above all the temperature gradient data. The dashed line is the envelope for the temperature gradient data and can be used for maximum daily positive lateral temperature gradient prediction through the given maximum daily solar radiation intensity on the lower web (Equation (12)).

(12) $$T_{\;\max\text{-}\mathrm{la}}^{+}+2d=0.01377I_{\max\text{-}\mathrm{lw}}+4.8752$$

## 6. Simulation of Temperature Field with Finite Element Method

### 6.1. Finite Element Model

**Figure 7:**
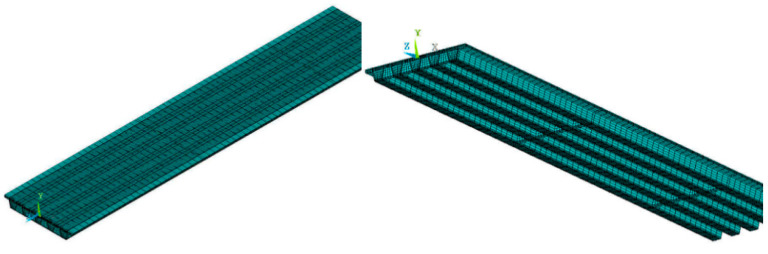
The 3D Finite Element model of the PC box girder.

A five-span continuous bridge model was created with ANSYS software. Since the bridge is symmetric in centerline, half of the five spans (2.5 spans) were modeled in ANSYS. The three-dimensional thermal element Solid 90 was selected for the structural analysis. The 3D model has 49,848 elements and 205,400 nodes, as shown in Figure 7.

### 6.2. Parameters Used in Finite Element Modeling

#### 6.2.1. The Thermo-Physical Properties of the Box Girder

Note that the asphalt pavement was built into the FE model together with the PC box girder. The thermo-physical properties for both of the asphalt and concrete are listed in Table 2.

#### 6.2.2. Boundary Conditions

**Figure 8:**
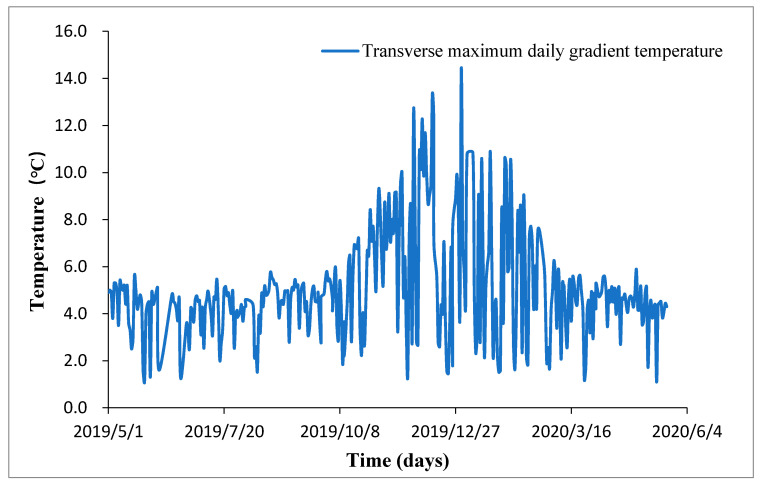
Maximum daily positive lateral temperature gradient variations.

It was observed during the one-year long monitoring, maximum daily positive lateral temperature gradient varied with solar altitude angle h. In summer, solar altitude angle h is larger than in winter, so that the solar radiation duration in the day is shorter leading to a smaller maximum daily positive lateral temperature gradient. It is evident in Figure 8 that December 31st witnessed the largest maximum daily positive lateral temperature gradient in the year.

**Figure 9:**
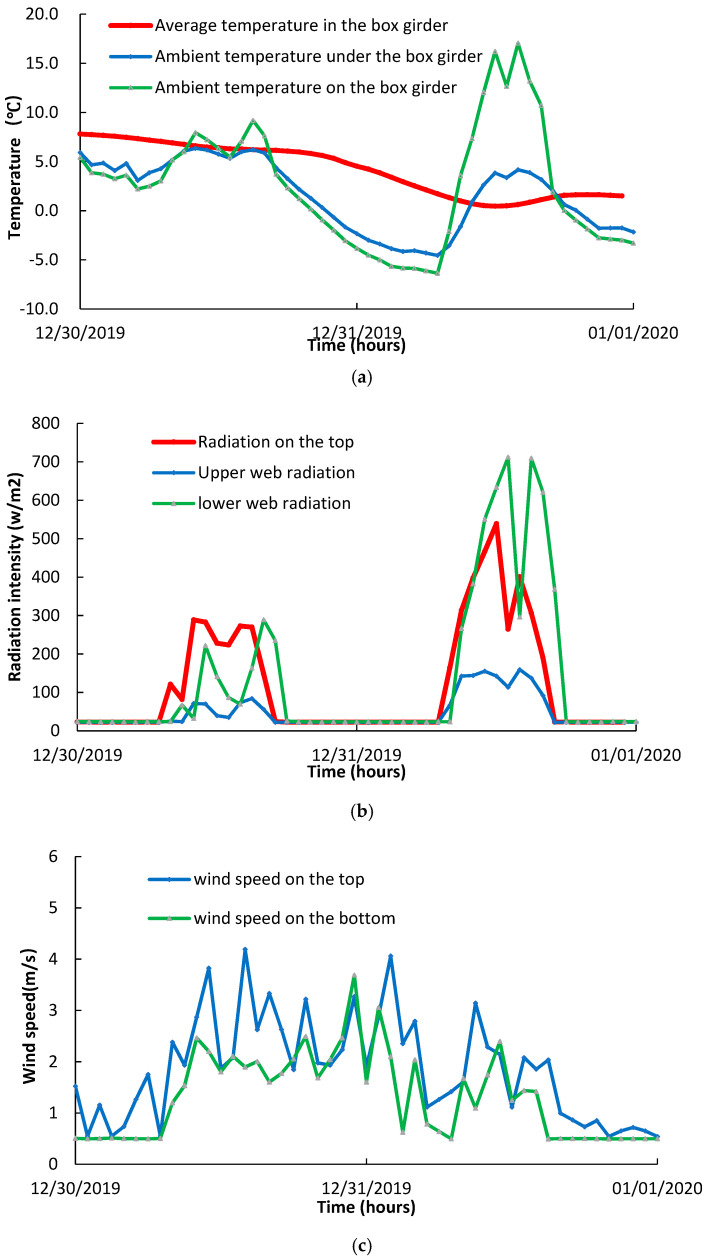
Variations on the boundaries of the box girder. (**a**) Temperature variations within and outside of the box girder; (**b**) solar radiation intensity variations on the top and web surface of the box girder on 30–31 December 2019; (**c**) wind speed variations on the top and web surface of the box girder on 30–31 December 2019.

Taking the monitoring data of the wind speed, solar radiation, and temperature within and out of the box girder on 30–31 December 2019 (Figure 9), it was observed that the ambient temperature on the top of the box girder and radiation intensity almost doubled on December 31 compared with that of December 30. As the ambient temperature increased on December 31, the outer surface of the web had been exposed to solar radiation longer in winter than the other time during the year. Meanwhile, the temperature within the box girder had less fluctuation and stayed low. Hence, the combining effects of the ambient temperature and solar radiation increase, and the not much changed inbox temperature, the maximum temperature gradient in the year, was generated in those days.

#### 6.2.3. Comparison between the FE Analysis and Experimental Results

**Figure 10:**
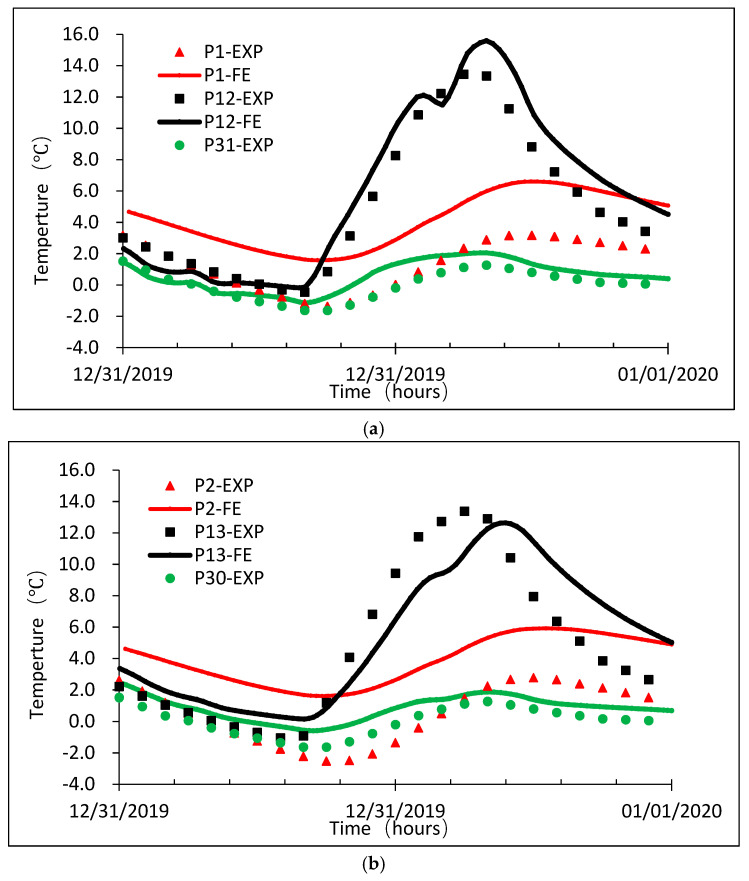

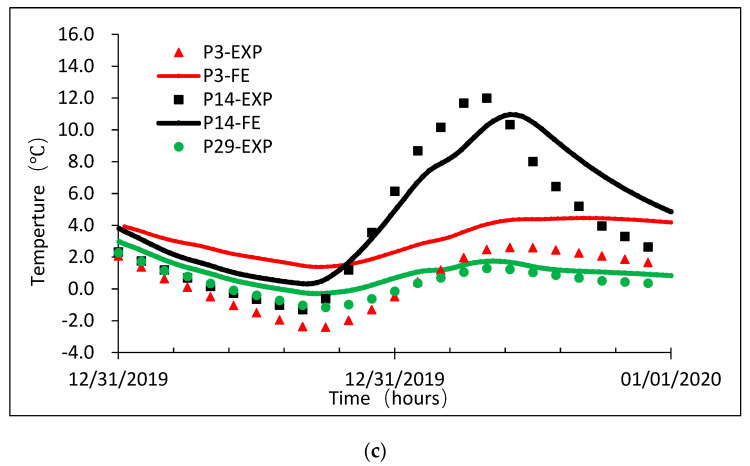
Measured and simulated temperature values comparisons on 31 December 2019. (**a**) Measured and simulated temperature values at the test point 1, 12, and 31 on 31 December 2019; (**b**) Measured and simulated temperature values at the test point 2, 13, and 30 on 31 December 2019; (**c**) Measured and simulated temperature values at the test point 3, 14, and 29 on 31 December 2019.

Figure 10 gives the comparison between the measured temperatures on 31 December 2019 and the simulated temperatures in FE analysis for nine temperature test points. The nine test points were grouped into three with each consisting one point on the top slab, one on the web, and one on the bottom slab. It can be seen from the comparison in Figure 10 that the differences between the measured and simulated temperatures are less than 2 °C except those on the top slab. The maximum measured and simulated temperature difference on the top slab reached 4.0 °C. Overall, the 3D FE model can be considered suitable for simulation of thermal conductivity, heat convection, ambient temperatures, and solar radiation in the box girder [6,8,11,14]. The difference between the measured and FE simulated temperature values may be attributed to the assumptions made in FE analysis that the heat thermal conductivity and the absorption rate are uniform in the structure. However, in really, those parameters could not be evenly distributed in heterogeneous materials like concrete and asphalt.

#### 6.2.4. Stresses Caused by the Spatial Temperature Gradients in FE Analysis

**Figure 11:**
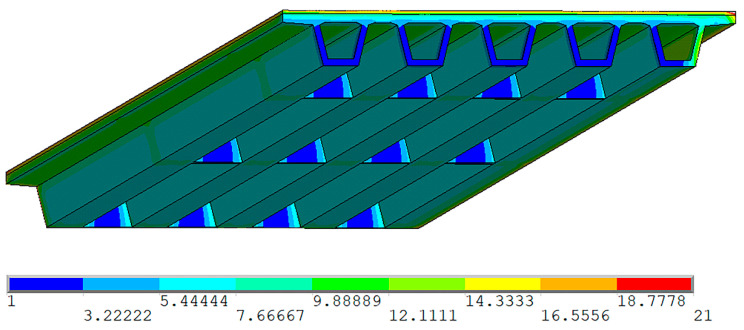
Simulated temperature distribution in the box girder at 15:00 on 31 December 2019 (unit: °C).

**Figure 12:**
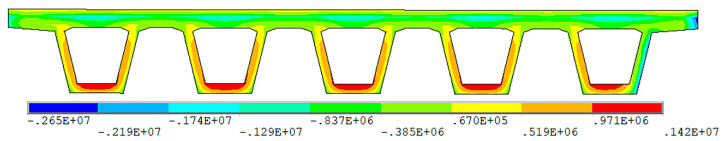
Longitudinal normal stress distribution under maximum positive lateral temperature gradient (unit: Pa).

**Figure 13:**
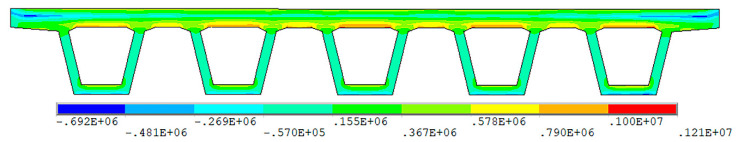
Transverse normal stress distribution under maximum positive lateral temperature gradient (unit: Pa).

Solar radiation causes differential temperature distributions that result in a vertical (*y* axis in Figure 7) and lateral (*x* axis in Figure 7) temperature gradient in the box girder. In bridge design practice, only the vertical temperature gradient is considered to be attributed to significant stress changes, and the lateral temperature gradient effect is generally ignored. In this paper, the lateral temperature gradient in the *x* axis direction is computed. Then, the maximum positive lateral temperature gradient was input in the FE model, and the corresponding normal stress in *z* axis direction and the transverse stress in *y* axis direct have been calculated, as shown in Figure 11, Figure 12 and Figure 13.

Figure 11 shows the temperature distribution at the time of 15:00 on 31 December 2019 when the lateral temperature gradient reached maximum. It is clear that the temperature on the top and right web surface reached maximum 21 °C, while the temperatures on the bottom surface and inside the box remain relatively low.

The positive lateral temperature gradient shown in Figure 11 was exerted onto the FE model for structural analysis and the longitudinal and transverse stress distributions due to the lateral temperature gradient were obtained. Figure 12 shows that the longitudinal normal stress reached maximum 2.65 MPa on the left web in compression and 1.42 MPa in tension on the other webs and bottom slabs. The longitudinal normal tensile stress caused by lateral temperature gradient alone is significant and large enough to create cracks in concrete. The transverse normal stress was also calculated, as shown in Figure 13. The bottom edge of the top slab experienced maximum transverse tensile stress 1.21 MPa, while the all the webs and bottom slabs were in compression, and the maximum compressive stress was 0.69 MPa. It is indicated that the maximum transverse tensile stress is comparable to the longitudinal one. Hence, the lateral temperature gradient effects cannot be ignored in structural design.

## 7. Conclusions

The authors performed an experimental study on temperature effects caused by solar radiation on a 5-span continuous PC box-girder bridge. Sensors measuring solar radiation, temperatures, strain, wind speed and displacements were installed at various cross sections. The continuously acquired data from May 2019 to May 2020 was utilized to determine the daily lateral maximum positive temperature gradient for the PC box-girder bridge. It was found from the data analysis that the Pearson’s correlation coefficient between the daily maximum positive lateral temperature gradient on the lower web and the daily maximum lateral solar radiation intensity reached maximum value of 0.879. Hence, the solar radiation on the web can be considered as the key factor that cause the daily maximum positive lateral temperature gradient. Then, a prediction equation for the daily maximum positive lateral temperature gradient was developed using solar radiation intensity measured on the lower web. Meanwhile, the comparison made between the simulated solar radiation intensities and the measured values indicated that there were differences between the simulated and measured values. It may be attributed to the fact that the uniform heat thermal transfer conductivity and absorption rate are used in the model. On the other hand, a linear elastic material model was utilized in the FE analysis for simplification, which may be attributed to causing the difference. In the future, an improved FE model accounting for the non-linear material properties, cracking, and stress re-distribution will be developed to generate more realistic stress distribution result. In addition, it is suggested that actual monitoring data be used to establish the temperature field boundary conditions when possible.

It was discovered that the daily maximum positive lateral temperature gradient took place at 15:00 on 31 December 2019 over the one-year monitoring. Entering the obtained daily maximum positive lateral temperature gradient into the FE model, the maximum longitudinal tensile stresses obtained were 1.42 MPa on the inner and farther webs and bottom slabs, and the maximum transverse ones were 1.21 MPa at the bottom of the top slab. The study demonstrated that the positive lateral temperature gradient effects were so significant that they should be taken into account in structural design.

Our future work will focus on developing an advanced three-dimensional solar radiation model accounting for modifications based on field testing data and comparing it with the vertical temperature gradient model in AASHTO bridge design specification.

## Figures and Tables

**Table 1:** Pearson correlation coefficients between daily maximum temperature gradient and daily maximum solar radiation.

Item	Daily Maximum Solar Radiation		
Position	Top slab (s1)	Upper web (s2)	Lower web (s3)
Pearson correlation coefficients	0.124	0.276	0.879

**Table 2:** The thermo-physical properties of the box girder and its asphalt pavement.

Material	Density (kg/m^3^)	Specific Capacity (J/kg °C)	Heat Thermal Conductivity (W/m °C)	Absorption Rate
Concrete	2500	880	2.5	0.4
Asphalt concrete	1700	1000	1.403	0.8
